# Angiomodulin (IGFBP7) is a cerebral specific angiocrine factor, but is probably not a blood–brain barrier inducer

**DOI:** 10.1186/s12987-020-00188-2

**Published:** 2020-04-01

**Authors:** Ofri Bar, Sivan Gelb, Kian Atamny, Shira Anzi, Ayal Ben-Zvi

**Affiliations:** Department of Developmental Biology and Cancer Research, The Institute for Medical Research Israel-Canada, Faculty of Medicine, Hebrew University of Jerusalem, 91120 Jerusalem, Israel

**Keywords:** Blood–brain barrier (BBB), Development, Angiomodulin (IGFBP7), VEGF-A, Angiocrine factors, Choroid plexus

## Abstract

**Background:**

Several secreted factors have been identified as drivers of cerebral vasculature development and inducers of blood–brain barrier (BBB) differentiation. Vascular endothelial growth factor A (VEGF-A) is central for driving cerebral angiogenesis and Wnt family factors (Wnt7a, Wnt7b and norrin) are central for induction and maintenance of barrier properties. Expressed by developing neural tissue (neuron and glia progenitors), they influence the formation of central nervous system (CNS) vascular networks. Another type of factors are tissue-specific paracrine factors produced by endothelial cells (ECs), also known as ‘angiocrine’ factors, that provide instructive signals to regulate homeostatic and regenerative processes. Very little is known about CNS angiocrine factors and their role in BBB development. Angiomodulin (AGM) was reported to be expressed by developing vasculature and by pathological tumor vasculature. Here we investigated AGM in the developing CNS and its function as a potential BBB inducer.

**Methods:**

We analyzed microarray data to identify potential angiocrine factors specifically expressed at early stages of barrier formation. We then tested AGM expression with immunofluorescence and real-time PCR in various organs during development, post-natal and in adults. Permeability induction with recombinant proteins (Miles assay) was used to test potential interaction of AGM with VEGF-A.

**Results:**

Several angiocrine factors are differentially expressed by CNS ECs and AGM is a prominent CNS-specific angiocrine candidate. Contrary to previous reports, we found that AGM protein expression is specific to developing CNS endothelium and not to highly angiogenic developing vasculature in general. In skin vasculature we found that AGM antagonizes VEGF-A-induced vascular hyperpermeability. Finally, CNS AGM expression is not specific to BBB vasculature and AGM is highly expressed in non-BBB choroid-plexus vasculature.

**Conclusions:**

We propose AGM as a developmental CNS vascular-specific marker. AGM is not a pan-endothelial marker, nor a general marker for developing angiogenic vasculature. Thus, AGM induction in the developing CNS might be distinct from its induction in pathology. While AGM is able to antagonize VEGF-A-induced vascular hyperpermeability in the skin, its high expression levels in non-BBB CNS vasculature does not support its potential role as a BBB inducer. Further investigation including loss-of-function approaches might elucidate AGM function in the developing CNS.

## Background

The central nervous system (CNS) requires a tightly regulated environment to provide the proper chemical composition for brain functions. This environment is maintained by the blood–brain barrier (BBB). The BBB is constructed of brain tissue-specific endothelial cells (ECs), which have a unique cell biology including sealing properties with specialized tight junctions and very low levels of transcytotic vesicles, and an array of transporters controlling substance influx and efflux [1–3].

For almost a century the medical and scientific community held a strong belief that the BBB is not functional in embryos or perinatal animals [2]. We and others found that the process of development of the BBB starts at early embryonic stages and that the BBB matures gradually to become functional for some of its properties even before birth [4, 5]. For example, we showed that the cerebral cortex BBB exhibits functional sealing properties by embryonic day 15.5 (E15.5) [4].

Comparing transcriptomes of peripheral ECs with those of CNS ECs allowed identification of molecular pathways that are drivers of cerebral vasculature development and inducers of BBB differentiation [4, 6, 7]. Secreted factors expressed by developing neural tissue (neuron and glia progenitors) influence the formation of CNS vascular networks by activating CNS-specific endothelial signaling pathways and transcriptional programs. These include vascular endothelial growth factor A (VEGF-A) signaling, which is central for driving cerebral angiogenesis, and the Wnt/beta-catenin/TCF-LEF pathway (Wnt7a, Wnt7b and norrin), which is central for induction and maintenance of barrier properties [8–12]. Other molecular factors such as sonic hedgehog (SHH), retinoic acid, GPR124 and reelin were also shown to contribute to these developmental processes [13–17]. Once the time-line of BBB-genesis was identified, we also compared transcriptomes of lung and cortical endothelial cells at E13.5, reasoning that such comparison would emphasize early molecular events of barrier genesis. Indeed, we showed that one CNS EC specific gene, *Mfsd2a*, is critical for barrier-genesis [4].

Another type of factors that might be involved in CNS-specific vascular development are tissue-specific paracrine factors produced by ECs and other vascular cells. These factors, also known as ‘angiocrine’ factors, provide instructive signals to regulate homeostatic and regenerative processes. They affect the vasculature itself, but also balance the self-renewal and differentiation of stem cells and orchestrate organ regeneration and tumor growth [18]. Some angiocrine factors determine the shape, architecture, size and patterning of regenerating organs. Their expression pattern is tissue-specific and they can be produced constitutively or be regulated by other angiogenic factors. Thus, angiocrine factors have a potential to influence CNS-specific vascular developments and maintenance. Nevertheless, very little is known about CNS angiocrine factors and in particular about their role in BBB development.

Angiomodulin (AGM) also known as IGF binding protein 7 (IGFBP7), is a secreted factor (stored in Weibel-Palade granules) [19] reported to be expressed by developing vasculature in general and to be upregulated by pathological tumor vasculature [20]. In the adult rat brain, AGM expression was induced after stroke, where it may be involved in sprouting angiogenesis [21]. Finally, AGM mutation in humans was linked to familial retinal arterial macro aneurysms [22], further suggesting a relevance to the function of CNS vasculature.

In vitro studies suggested that AGM antagonizes VEGF-A-induced angiogenesis. AGM treatment suppresses VEGF-A-induced tube formation, cell migration and proliferation, but does not induce apoptosis [23, 24]. AGM binds to collagen type IV and other extracellular matrix or basement membrane-associated factors and also to VEGF-A itself [25]. There is also evidence supporting alternative interactions. Knockdown of both VEGF-A and AGM together revealed a synergistic effect on angiogenesis in zebrafish embryos, thus AGM interaction with the VEGF-A pathway seems essential for proper patterning of nascent vessels [20]. Finally, another study showed that AGM was strongly induced in ECs by VEGF-A, integrin (alpha-v beta-3) is a receptor for AGM, and that just like VEGF-A, AGM might increase vascular permeability [25].

Here, we analyzed microarray data to identify potential angiocrine factors specifically expressed at early stages of barrier formation. We identified the temporal and spatial expression of AGM in embryos during CNS angiogenesis and BBB formation. Contrary to previous reports, we found that AGM protein expression was specific to developing CNS endothelium and not to highly angiogenic developing vasculature in general. We used permeability induction with recombinant proteins (Miles assay) to test potential interaction of AGM with VEGF-A, and showed that AGM antagonized VEGF-A-induced vascular hyperpermeability. Finally, CNS AGM expression was not specific to BBB vasculature and it had high expression levels in non-BBB choroid-plexus vasculature, diminishing the hypothesis that it is involved in induction of BBB properties.

## Methods

### Mice

ICR mice were obtained from Envigo (Rehovot, Israel). Pregnant mice were obtained following overnight mating (day of vaginal plug is defined as embryonic day 0.5). All mice were bred and maintained in the animal facility of the Hebrew University under specific pathogen-free conditions. For all experiments male ICR mice were tested other than embryos in which both males and females were tested. All animals were treated according to institutional guidelines approved by the Institutional Animal Care and Use Committee (IACUC) at Hebrew University.

### Tissue preparation

After dissection, brains/head/peripheral organs were placed in 4% paraformaldehyde (PFA, Sigma Aldrich) at 4 °C overnight, cryopreserved in 30% sucrose and frozen in TissueTek OCT (Sakura). Frozen brains were cut to 12 µm slices for immunofluorescence staining (CM1950, Leica) to produce coronal brain sections.

### Immunofluorescence

12 µm thick cryo-sections were washed with phosphate buffered saline (PBS) for 5 min at room temperature (RT) and then incubated for 1 h at RT with blocking solution (10% bovine serum albumin (BSA), 10% normal horse serum (NHS), 0.5% triton X-100 in PBS). Slides were incubated with primary antibodies (diluted in 2.5% BSA, 2.5% NHS, 0.5% triton X-100 in PBS) at 4 °C overnight. Slides were then washed with PBS, incubated with secondary antibodies for 1 h at RT, washed and mounted with 4′,6-diamidino-2-phenylindole (DAPI) Fluoromount-G (Southern Biotech). All sections being compared in different tissues and in the same tissue at different ages were incubated under identical conditions and at the same time. For every staining, we included a condition with only secondary antibodies in order to define secondary non-specific tissue binding.

### Primary antibodies used

Hamster anti-mouse CD31 (1:100, Bio-Rad cat No. MCA1370Z), mouse anti-mouse NeuN (1:50, Millipore cat No. MAB377), mouse anti-mouse S100b (1:400, abcam cat No. AB11178), rat anti-mouse CD45 (1:100, BioLegend cat No. 103102), rat anti-mouse PDGFRb (1:100, Invitrogen cat No. 14140282), and polyclonal rabbit anti-mac25/AGM (1:100), which was a kind gift from Prof. Masayuki Miyasaka (Laboratory of Molecular and Cellular Recognition, Department of Internal Medicine and Molecular Science, Osaka University Graduate School of Medicine, Japan) [26]. AGM antibody production and characterization as described by the authors [26] in brief: Full-length mouse mac25/AGM cDNA (GenBank AB012886) was cloned into baculovirus expression system. Secreted recombinant mac25/AGM with a molecular size of ~ 34 kDa was purified by affinity chromatography with the Ni^2+^-based Probond resin (Invitrogen) and gel filtration chromatography (Sephadex-G200; Amersham, Little Chalfont, UK), and finally concentrated by ultrafiltration (CentriPlus YM-10; Millipore, Bedford, MA). The N-terminal sequence of the purified protein (Ser–Ser-Ser-Asp-Ala-Cys-Gly-Pro-Cys-Val) was found to be identical to that of mature mouse mac25/AGM, indicating that it was a mature full-length form of mac25/AGM. pAb was raised against the recombinant mac25/AGM protein by subcutaneous immunization of New Zealand White female rabbits with the purified recombinant mac25/AGM (0.2 mg) emulsified in complete Freund’s adjuvant. The polyclonal IgG was affinity purified from the immunized serum using a Protein G ± Sepharose CL-4B (Amersham BioSciences) column and a recombinant mac25/AGM-conjugated column. The affinity-purified pAb successfully detected the 34-kDa recombinant mac25/AGM protein on and the anti-mac25/AGM pAb the endogenous mac25/AGM produced by bEnd.3 cells on Western blots. The antibody failed to react against human mac25/AGM that has high sequence homology (87%) to mouse mac25/AGM.

### Secondary antibodies used

Cy3-goat anti-Armenian hamster (1:500, Jackson cat No. 127165160) and Alexa 488 donkey anti-rabbit IgG (1:200, Jackson cat No. 711545152), Alexa 647 donkey anti-rat IgG (1:200, Jackson cat No. 712605153), Cy3 donkey anti-mouse IgG (1:500, Jackson cat No. 715165151).

### Fluorescence microscopy

Images presented were taken using Olympus BX51, 10X/0.3 and 20X/0.5, with Andor Zyla CCD camera, Nikon NIS elements software (version D4.5) for both image acquisition and analysis. High magnification images (lower panel Fig. 2 and lower panel Additional file 1: Figure S2) were captured using Nikon Eclipse Ni confocal microscope, objective ×40 with Nikon C2 camera and Nis-Elements software. Images are maximal z-projection of optical sections taken from a 12 µm tissue section imaged with 0.85 µm intervals.

### Miles vascular permeability assay

The assay was carried out as in Zhang et al. [27] 8 weeks old male ICR mice backs were shaved a day prior to injections. Deeply anesthetized (85% Ketamine, 15% Xylazine), mice were injected intravenously with Evans blue dye (sigma e2129, 200 µl of 0.5% solution in saline (0.9%NaCl)). Following 10 min circulation, 50 µl recombinant proteins (VEGF-A (#100-20 PeproTech, Rehovot, Israel) and AGM (#350-09 PeproTech, Rehovot, Israel) 250 pg/ml dissolved in saline) were injected sub-dermally in designated spots on the mouse backs. In each mouse, we injected four spots; VEGF-A, AGM, combined VEGF-A + AGM and saline. Following 30 min, the animals were euthanized and the skin area including the entire injection site was removed by punch biopsies and placed in 2 ml of formamide (Merck) at room temperature for 48 h. The optical absorption of the biopsy elution samples was measured at 595 nm using 96 well microplate reader (Tecan, infinite 200 pro). Each biopsy elution was measured in quadruplicates, blank measurement was omitted and the average absorption was calculated. For each mouse, the permeability factor was calculated as fold increase over the negative control saline sample. We presented the average permeability factor for each condition of four mice (data from mice that VEGF-A (as the positive control) did not induce hyperpermeability were excluded).

### Microarray

Data analyzed in the current study were produced from our previous study [4] according to methodology described in our published manuscript (Ben-Zvi et al. [4]). In brief: homozygous Tie2-GFP transgenic mice (Jackson laboratory, strain 003658) were used for BBB transcriptional profiling. E13.5 Tie2-GFP embryos were micro-dissected for cortex and lungs. Cortex tissue was carefully cleared of the meninges and choroid plexus. FACS purification of GFP-positive cells and GeneChip analysis was performed. RNA was purified with Arcturus PicoPure RNA isolation kit (Applied biosystems), followed by NuGEN Ovation V2 standard linear amplification and hybridization to Affymetrix Mouse Genome 430 2.0 Array. All material from a single litter (10–13 embryos) was pooled and considered as a biological replicate. Four biological replicates were used. Each biological replicate represents purification from different litters performed on different days. Data is available in the NCBI’s Gene Expression Omnibus (http://www.ncbi.nlm.nih.gov/geo/), accessible through GEO series accession number GSE56777. Presented values are average of four biological repetitions.

### Real time PCR analysis

Forebrain tissue was carefully cleared of meninges and choroid plexus (lung and heart tissue was dissected from the same mice/embryos) and total RNA was purified using BIO TRI RNA reagent (bio-lab). Total RNA (2 μg) was used for first-strand cDNA synthesis using random primers and reverse transcriptase (Applied Biosystems). Real-time PCR was performed with SYBR Green master mix (Applied Biosystems) in 384-well plates using the c1000-bio-rad thermo cycler CFX384 real time system. All reactions were performed in triplicates with four-five biological replicates. The relative amount of mRNA was calculated using the comparative CT method after normalization to CD31. The following primers were used: Mouse CD31 Forward primer 5′-CTCACGCTGGTGCTCTATG-3′ and reverse primer 5′-CCATTCATCACCTCCCATGAT-3′. Mouse AGM forward primer 5′-CCACGAGCACCTTGTTCA-3′ and reverse primer 5′-TGTCATCTCTGGGCTCTCA-3′.

### Statistical analysis

Sample size for all immunofluorescence experiments was determined empirically using standards generally employed by the field: a minimum of three animals per group in each experiment and a minimum of four tissue sections of each tissue. Standard error of the mean was calculated for all experiments and displayed as errors bars in graphs. Statistical details for specific experiments can be found in the figure legends. For the Miles assay data and microarray data, Mann–Whitney U test (non-normally distributed data) was used for two-group comparisons. For the qPCR data, two group comparisons were analysed using an unpaired two-tailed Student’s t test; P < 0.05 was considered significant.

## Results

### Angiocrine factors are differentially expressed by cerebral endothelium at early stages of BBB induction and differentiation

**Fig. 1:**
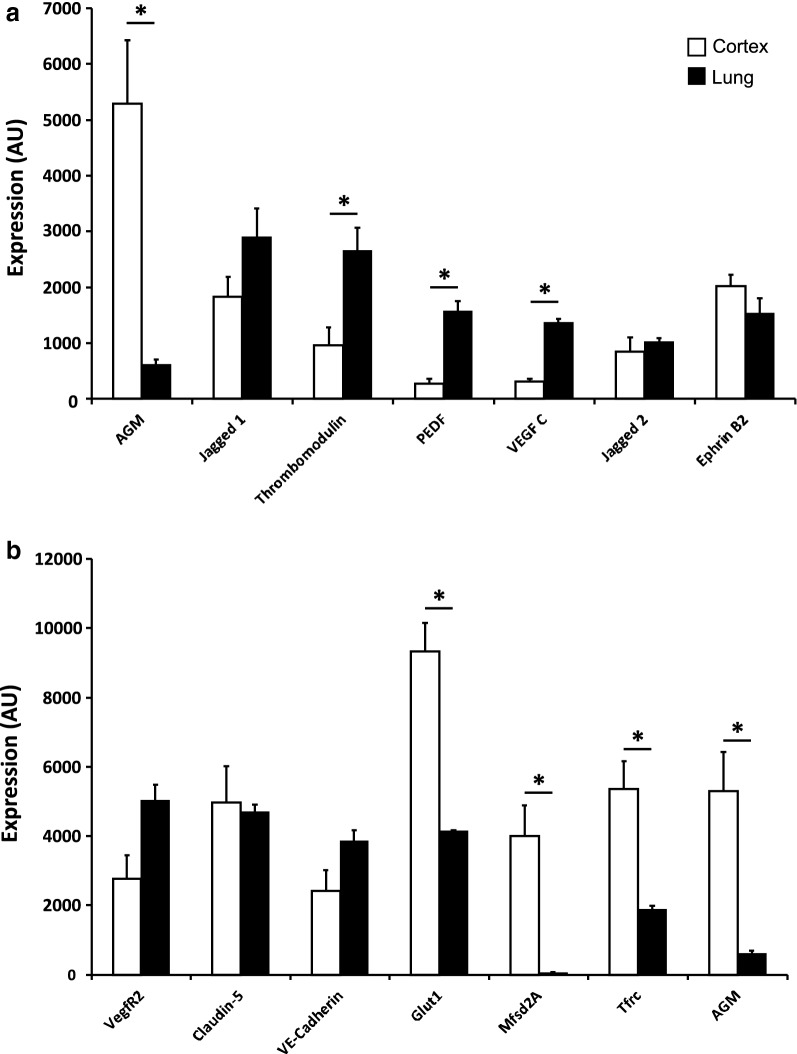
Angiocrine factors are differentially expressed by cerebral endothelium at early stages of BBB induction and differentiation. Microarray data analysis from our previous study [4] comparing transcriptomes of lung and cortical endothelial cells at E13.5 of potential angiocrine factors, previously suggested to be involved in CNS vasculature biology [18]. **a** Some angiocrine factors showed similar transcripts levels in both lung and cortex ECs (Jagged2 and EphrinB2). Other angiocrine factors showed higher levels in lung ECs, of which thrombomodulin, pigment epithelium-derived factor (PEDF) and VEGF-C were statistically significant (P < 0.05). AGM transcripts levels were significantly higher in cortex ECs then in lung ECs (more than eightfold increase, P < 0.05). **b** Transcripts level of AGM in cortex ECs was comparable to levels of the most highly expressed BBB genes (Glut1, Tfrc, Mfsd2a). Statistically significant differential transcripts levels (P < 0.05) are unique to BBB-enriched markers. Pan-endothelial markers (VegfR2, Claudin-5 and VE-Cadherin) show similar levels in both lung and cortex ECs at this stage. N = 4 mice. All data are mean ± sem. Statistical significance determined by a non-parametric Mann–Whitney U test

We analyzed microarray data from our previous study [4] that compared transcriptomes of lung and cortical endothelial cells, both at E13.5 to identify potential angiocrine factors specifically expressed at early stages of barrier formation. While many secreted factors were found to be differentially expressed, we focused on angiocrine factors that were previously suggested to be involved in CNS vasculature biology [18]. Some angiocrine factors showed similar transcript levels in both lung and cortex ECs (Fig. 1a, e.g. Jagged2 and EphrinB2). Other angiocrine factors showed higher levels in lung ECs, of which differences in expression levels where statistically significant for thrombomodulin, pigment epithelium-derived factor (PEDF) and VEGF-C (Fig. 1a). AGM transcript levels were significantly higher in cortex ECs then in lung ECs (more than eightfold increase). Moreover, transcript levels of AGM in cortex ECs was comparable to levels of the most highly expressed genes, which are most commonly used as BBB markers (Fig. 1b. Glut1, Tfrc, Mfsd2a). Such differential transcript levels are unique to BBB-enriched markers as pan-endothelial markers show similar levels in both lung and cortex ECs at this stage (Fig. 1b, VegfR2, Claudin-5 and VE-Cadherin). These findings led us to consider AGM as a prominent candidate that might play a role in BBB formation. We therefore sought to validate AGM expression at the protein level and investigate the specific pattern of its expression during embryonic BBB formation.

### Angiomodulin protein expression is specific to developing cerebral vasculature and not to highly angiogenic developing vasculature of peripheral organs

**Fig. 2:**
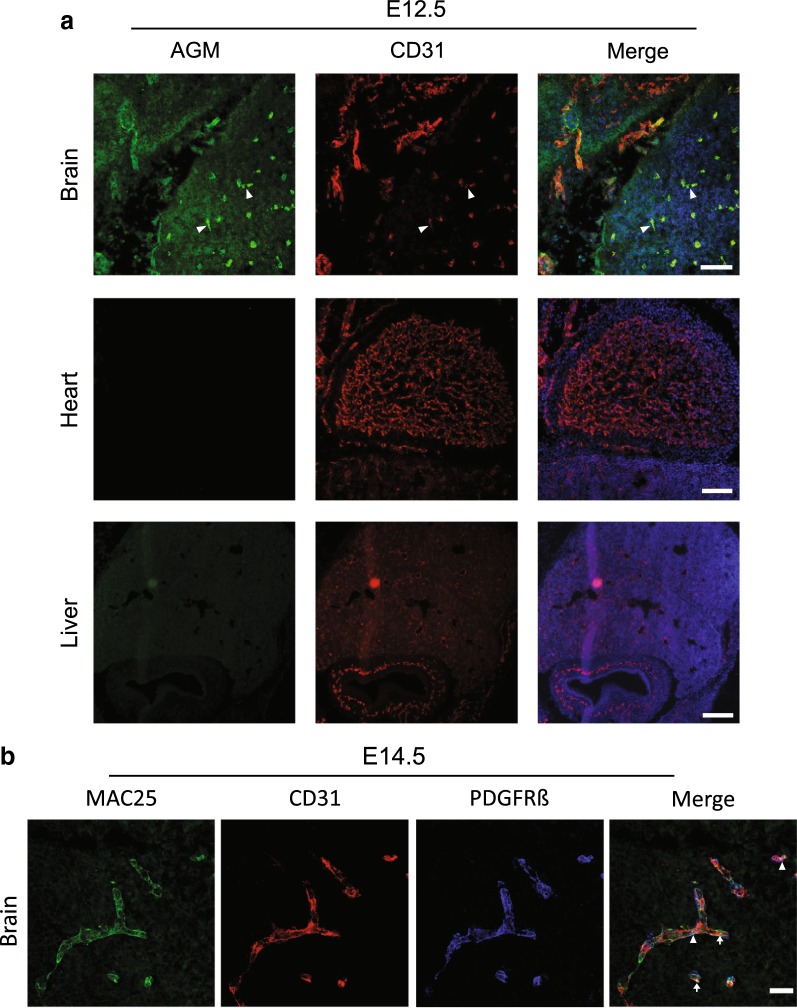
Angiomodulin protein expression is specific to developing cerebral vasculature and not to highly angiogenic developing vasculature of peripheral organs. **a** AGM (green) could be detected as early as E12.5 in cortical vasculature (upper panel, arrow heads), co-labeled with CD31 positive endothelium (red). AGM expression was specific to CNS vasculature, as it was not detected in developing peripheral organs such as the heart or liver (middle and lower panels). Merge images includes DAPI nuclei staining. N = 3 mice; scale bar 100 µm for two upper panels and 200 µm for the lower panel. **b** AGM (green) co-localizes with CD31 positive endothelial cells (red, arrows) and PDGFR-beta pericytes (blue, arrow heads) shown in high magnification confocal imaging of E14.5 cortical vasculature. Scale bar 25 µm

We tested AGM protein expression with immunofluorescence using a polyclonal rabbit anti-mac25/AGM [26] in various organs during development. We validated our microarray findings at the protein level; AGM could be clearly detected as early as E12.5 in cortical vasculature (Fig. 2, upper panel, arrow heads). AGM expression was specific to CNS vasculature, as it was not detected in developing peripheral organs such as the heart or liver (Fig. 2, middle and lower panels). At this developmental stage, CD31 positive vascular networks could be noticed in these peripheral organs, which are negative to AGM. In the brain vasculature, AGM co-localized with both endothelial cells and pericytes (Fig. 2, E14.5 forebrain vessels in high magnification). We concluded that contrary to previous reports, at least according to our AGM antibody staining, AGM expression is not a general characteristic of highly active angiogenic vasculature in the developing mouse embryo but rather a marker of CNS active angiogenic vasculature.

### Angiomodulin antagonizes VEGF-A-induced peripheral vascular hyperpermeability

**Fig. 3:**
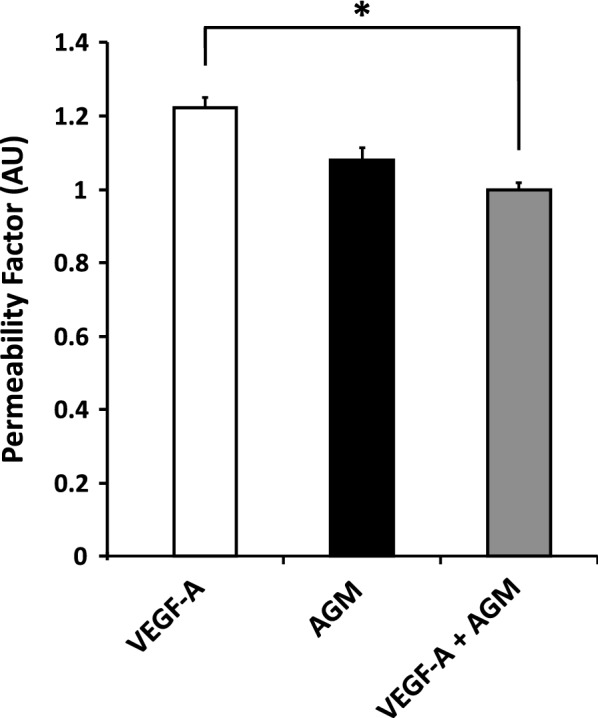
Angiomodulin antagonizes VEGF-A-induced peripheral vascular hyperpermeability. AGM’s effect on VEGF-A-induced vascular permeability was tested with the Miles assay. Recombinant VEGF-A and AGM proteins were injected sub-dermally to mice that received Evans-Blue tracer injection intravenously. Skin biopsy was taken from the injection site. The tracer was extracted and tracer levels were quantified with spectrophotometric absorbance. Relatively low VEGF-A concentrations (250 pg/ml) induced approximately 20% increase in vascular hyperpermeability over control (saline). Relatively low AGM concentrations (250 pg/ml), did not induced significant permeability. Injections of VEGF-A together with AGM, abolished VEGF-A-induced hyperpermeability (differences in leakage level between VEGF-A and the combined VEGF-A/AGM was statistically significant P < 0.05). Data represents permeability fold induction over saline control (indicated as the Permeability Factor). N = 4 mice. All data are mean ± sem. Statistical significance determined by a non-parametric Mann–Whitney U test

Having confirmed AGM-specific expression in CNS endothelium at early stages of barrier-genesis, we turned to testing the hypothesis that AGM might be a negative modulator of VEGF-A-induced permeability. VEGF-A is expressed by the avascular neural tube to induce vascular sprouts ingression from the perivascular neural plexus, which surrounds the neural tube, into the CNS parenchyma. VEGF-A is necessary for inducing proper vascular tube formation as well as for endothelial survival. At later stages, once vessels are maturing and stabilizing, they no longer depend on VEGF-A for their survival [28]. Interestingly, this maturation process is synchronized with the CNS vasculature’s acquisition of BBB properties [28]. VEGF-A is also responsible for inducing vascular hyperpermeability. For example, it is known to induce and maintain fenestration in liver vasculature [29] and to induce leakage of blood born tracers upon exposure to skin vasculature. Therefore, in developing CNS vasculature, potential VEGF-A induction of non-barrier properties should somehow be modulated in order to allow proper induction of angiogenesis together with induction of barrier-genesis. To test AGM’s effect on VEGF-A-induced vascular permeability, we used the Miles assay: recombinant VEGF-A and AGM proteins were injected sub-dermally to mice that received Evans-Blue tracer injection intravenously. Skin biopsy was taken from the injection site (half an hour post-exposure). The tracer was extracted and tracer levels were quantified with spectrophotometric absorbance (Fig. 3). Relatively low VEGF-A concentrations were used (250 pg/ml) to induce minimal hyperpermeability. As expected, VEGF-A induced approximately 20% increase in vascular hyperpermeability over control (saline), which was statistically significant. At relatively low AGM concentrations (250 pg/ml), AGM by itself did not induced significant permeability. Once VEGF-A was injected together with AGM, VEGF-A, induced hyperpermeability induction was abolished (differences in leakage level between VEGF-A and the combined VEGF-A/AGM was statistically significant, whereas differences in leakage level between the combined VEGF-A/AGM and AGM were insignificant). Therefore at least in skin vasculature, AGM might act as a negative modulator of VEGF-A-induced permeability.

### Angiomodulin protein expression in adult mouse brain is not restricted to the vasculature

**Fig. 4:**
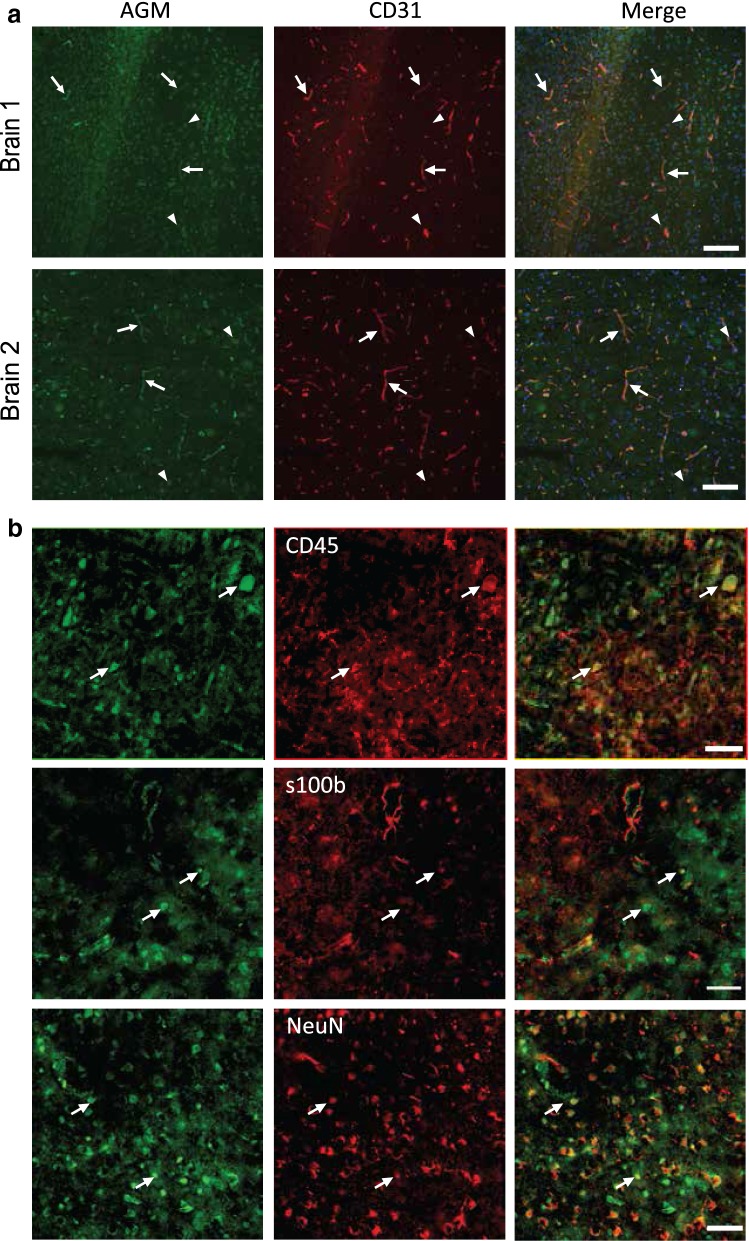
Angiomodulin protein expression in adult mouse brain is not restricted to the vasculature. **a** AGM (green) is expressed in adult CNS vasculature (examples of representative cortical sections form two adult mice). Different from the embryonic situation, in addition to vasculature staining (arrows, co-labeled with CD31 positive endothelium (red)), we also detected cellular staining in CNS parenchyma (arrow heads). Merge images includes DAPI nuclei staining. N = 3 mice; scale bar 100 µm. **b** AGM staining in CNS parenchyma includes co-localization with a subset of microglia cells (labeled with CD45, arrows upper panel), co-localization with a subset of astrocytes (labeled with S100-beta, arrows middle panel) and co-localization with a subset of neurons (labeled with NeuN, arrows lower panel). Scale bar 50 µm

A previous study characterized AGM’s pattern of expression in adults with a knock-in mouse (AGM^lacZ/+^) [20]. According to these findings, β-gal reporter activity was predominantly localized to the SMC-invested large vessels with lesser expression in capillaries of all organs. Only when a high sensitivity β-gal protocol was used, AGM expression could be indicated in all adult vasculature (including the brain), except liver and bone marrow where it was expressed at low levels [20]. These findings might suggest differential AGM expression levels similar to the embryonic situation, were CNS capillaries show higher levels of AGM than peripheral vasculature (Fig. 2). Adult CNS capillaries are refractory to VEGF-A-induced hyperpermeability and thus maintenance of AGM expression in adults might mediate this effect. Using immunofluorescence of adult mouse cortex, we found that AGM is indeed expressed in CNS vasculature (Fig. 4a). We could not detect AGM expression in capillaries of peripheral organs of adult mice; in the heart we could detect some AGM staining in large vessels, and in the liver low AGM staining could be detected in a small fraction of non-vascular cells (presumably hepatocytes, arrowheads Additional file 1: Figure S1). Different from the embryonic situation, in addition to vasculature staining, in adults we also detected cellular staining in CNS parenchyma (Fig. 4). AGM staining in CNS parenchyma includes co-localization with a subset of CD45 positive microglia cells, a subset of S100-beta positive astrocytes and a subset of NeuN positive neurons (Fig. 4b). Therefore, in adult CNS, AGM should not be used as a CNS endothelial-specific marker.

### Cerebral angiomodulin protein expression is not specific to BBB vasculature and it is highly expressed in the choroid plexus vasculature

**Fig. 5:**
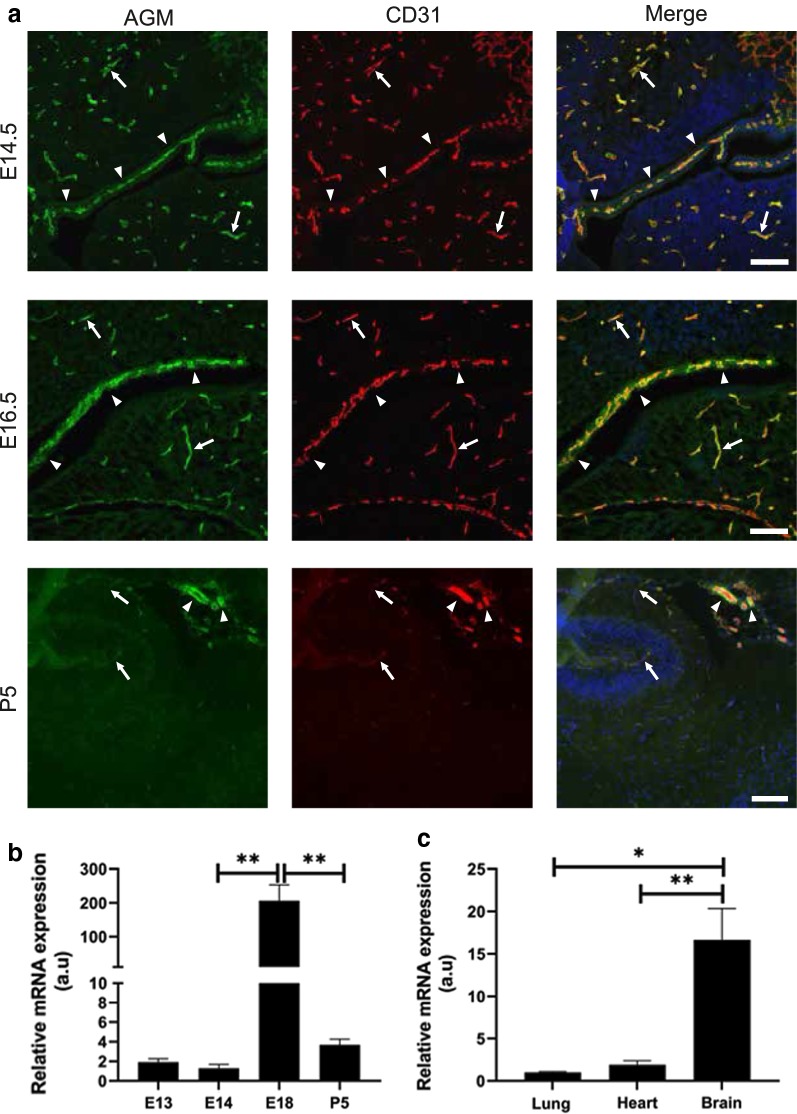
Cerebral Angiomodulin protein expression is not specific to BBB vasculature and it is highly expressed in the choroid plexus vasculature. **a** At E14.5–E16.5, AGM (green) was expressed in CNS vasculature (arrows), but this expression was not restricted to BBB-forming vessels as it was also seen in highly permeable vessels of the choroid plexus (arrow heads). This pattern persisted into post-natal stages; at P5 (lower panel) we detected lower AGM levels at cortical BBB vessels in comparison to the persistent high expression in choroid plexus vessels. Choroid plexus AGM expression was detected in all brain ventricles as demonstrated for lateral ventricles (upper panel—E14.5 and middle panel—E16.5) and for third ventricle (lower panel—P5, arrow heads). N = 3 mice; scale bar 100 µm. **b** Forebrain AGM mRNA levels were evaluated with real-time PCR along embryonic and post-natal development (normalized to CD31 mRNA levels). In the current analysis, the highest AGM levels were detected at E18.5 (P = 0.002). N = 5 mice. **c** At E18.5, forebrain AGM mRNA levels were significantly higher (~ 16-fold) than lung (P = 0.01) or heart (P = 0.004). N = 4 mice. All data are mean ± sem. Statistical significance determined by an unpaired two-tailed Student’s t test

In order to determine when during development AGM expression in the CNS shifts from being vasculature-specific to also being expressed in other cell types, we stained cortical tissues at various developmental and post-natal stages. At E14.5-E16.5, AGM was specifically expressed in CNS vasculature (Fig. 5, arrows), but this expression was not restricted to BBB-forming vessels as it was also seen in highly permeable vessels of the choroid plexus (Fig. 5, arrow heads). This pattern persisted into post-natal stages; at P5 we detected lower AGM levels at cortical BBB vessels in comparison to the persistent high expression in choroid plexus vessels. Choroid plexus AGM expression was detected in CP of all brain ventricles as demonstrated for lateral ventricles (Fig. 5, upper (E14.5) and middle (E16.5) and for third ventricle (Fig. 5, lower panel (P5), arrow heads) as well as in forth ventricle CP (data not shown). AGM expression in non-vascular CNS cells is therefore apparent only in adults. As in E12.5, AGM expression was also specific to CNS vasculature in E14.5, as it was not detected in developing peripheral organs such as the heart, liver or lung (Additional file 1: Figure S2). We concluded that as development progresses from embryonic stages E14.5 on, AGM is expressed in all CNS vasculature including choroid plexus vasculature, and therefore should not be considered as a BBB specific marker at these stages.

## Discussion

CNS endothelium expresses AGM throughout different stages of development. Here, we show CNS endothelium expression in embryonic and adult stages and therefore AGM could be considered as a CNS-specific angiocrine factor. Only at an early stage of development (E12.5), is AGM specifically expressed in BBB-forming endothelial cells. By E14.5, AGM expression is additionally found in the choroid plexus vasculature, which has non-BBB vascular properties, and as the embryonic development progresses, AGM staining in BBB vasculature weakens. This finding led us to the conclusion that only at early stages of development, can AGM reliably be used as a specific BBB marker. In the brain we could not detect major differences in vascular AGM staining of different brain regions. During embryonic and post-natal stages, AGM expression is restricted to CNS endothelium and is not expressed in capillaries of peripheral organs.

Although AGM was reported to be up regulated during angiogenesis in pathological conditions and in all organs during embryonic development where angiogenesis is pronounced [20], according to our findings its expression is not detectable in developing peripheral embryonic endothelium during angiogenesis and it is restricted to CNS vasculature. In fact, the reported whole mount β-gal reporter staining of a knock-in mouse (AGM^lacZ/+^) [20] can also be interpreted as demonstrating CNS specific expression (in forebrain and spinal cord) rather than pan-vascular embryonic expression (Figure S2 of Hooper et al. [20]).

In the adult mouse CNS, AGM expression is not specific to the endothelium and can be detected in other cortical parenchymal cells. This finding is in line with recent mRNA profiling that demonstrated detectable AGM transcripts also in, vascular smooth muscle cells, vascular-associated fibroblasts and pericytes [30]. AGM mRNA levels are relatively high and do not differ dramatically between the different ECs in the vascular tree (slightly lower in venous ECs than in capillary and arterial ECs, but still relatively high) [30]. According to mRNA profiling in another study, AGM mRNA levels are also relatively high in ECs and mature astrocytes, and detectable in neurons also in human brains [31]. AGM expression such as that found by us in non-BBB vasculature at the choroid plexus is also in line with mRNA profiling of the embryonic and adult choroid plexus [32].

The molecular programs that govern the production of context-dependent angiocrine factors from organ-specific ECs remain largely undefined. AGM expression was found to be down regulated by promoter hypermethylation in prostate cancer [33]. Based on genome-wide methylation profiling of developing BBB ECs, we found some supporting data of progressive de-methylation in genomic sites associated with AGM, which might indicate an epigenetic control mechanism of AGM expression levels (data not shown). Based on in vitro studies, AGM expression is also induced by VEGF-A [25] and therefore it is possible that there is a molecular negative feedback loop in which up regulation of AGM levels in response to VEGF-A then antagonizes VEGF-A function.

VEGF-A signaling orchestrates CNS angiogenesis. Paradoxically, VEGF-A is also a strong inducer of non-BBB properties and therefore, potential VEGF-A induction of non-barrier properties should somehow be modulated in order to allow proper induction of angiogenesis together with induction of barrier-genesis. This led us to the hypothesis that AGM might be a negative modulator of VEGF-A-induced permeability, especially in light of the reported direct binding of AGM to VEGF-A [25]. There are conflicting interpretations for experimental investigations of VEGF-A and AGM interactions. While AGM antagonizes VEGF-A-induced angiogenesis in vitro [23], knockdown of both VEGF-A and AGM together revealed a synergistic effect on angiogenesis in zebrafish embryos [20]. Finally, just like VEGF-A, AGM (at relatively high concentrations of 1–10 µg/ml) might increase vascular permeability [25]. Based on our skin permeability data (Fig. 3), we suggest that AGM might have an antagonizing effect on VEGF-A induced permeability. It is possible that high levels of AGM (shown previously to induce hyperpermeability) corresponds to the expression levels observed by us at the choroid plexus vasculature and that low levels of AGM (that antagonize VEGF-A induced permeability) correspond to the lower expression levels at CNS BBB capillaries. Accordingly, loss of AGM expression in cortical vasculature is predicted to perturb BBB-genesis and loss of AGM expression in the choroid plexus is predicted to reduce permeability in these vessels. Further investigation including loss-of-function approaches might elucidate AGM function in the developing CNS.

## Conclusions

We propose that AGM is a developmental CNS vascular-specific marker. AGM is not a pan-endothelial marker, nor a general marker for developing angiogenic vasculature. Thus, AGM induction in the developing CNS might be distinct from its induction in pathology. While AGM is able to antagonize VEGF-A-induced vascular hyperpermeability in the skin, its high expression levels at non-BBB CNS vasculature do not support its potential role as a BBB inducer.

## Supplementary information

**Additional file 1: Figure S1.** Absence of AGM expression in capillaries of peripheral organs of adult mice. In the heart (upper panel) we could detect some AGM staining (green) only in large vessels, and in the liver (lower panel), low AGM staining could be detected in a small fraction of non-vascular cells (presumably hepatocytes, arrowheads) but not in the vasculature. N = 3 mice; scale bar 50 µm. **Figure S2.** Absence of AGM expression in capillaries of developing peripheral organs. As in E12.5, AGM expression was specific to CNS vasculature also in E14.5, as it was not detected in developing vasculature of peripheral organs such as the heart, liver or lung. N = 3 mice; scale bar 100 µm (upper and middle panel) and 50 µm (lower panel).

## Data Availability

The microarray data analyzed in the current study are available in the NCBI’s Gene Expression Omnibus (http://www.ncbi.nlm.nih.gov/geo/) and are accessible through GEO series Accession number GSE56777.

## Abbreviations

AGM: Angiomodulin
IGFBP7: IGF binding protein 7
VEGF-A: Vascular endothelial growth factor A
CNS: Central nervous system
ECs: Endothelial cells
BBB: Blood brain barrier
