# Supplementary figures and images for: Angiomodulin (IGFBP7) is a cerebral specific angiocrine factor, but is probably not a blood–brain barrier inducer
Source: Fluids Barriers CNS. 2020 Apr 1;17:27. doi: 10.1186/s12987-020-00188-2 (PMC7110827; doi:10.1186/s12987-020-00188-2)

# Additional File 1 : Figure S1

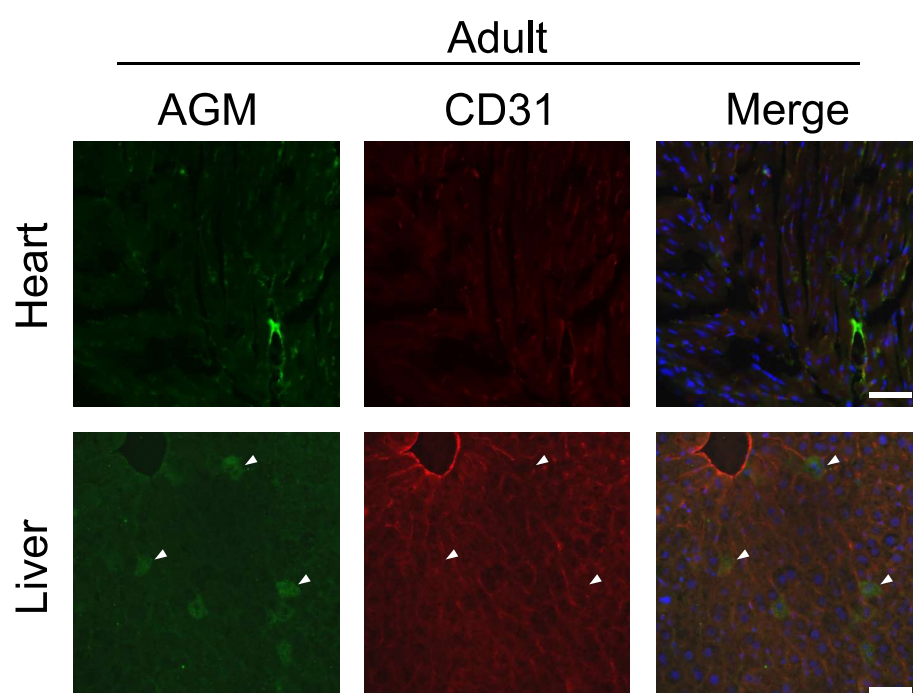

## Additional File 2 : Figure S2

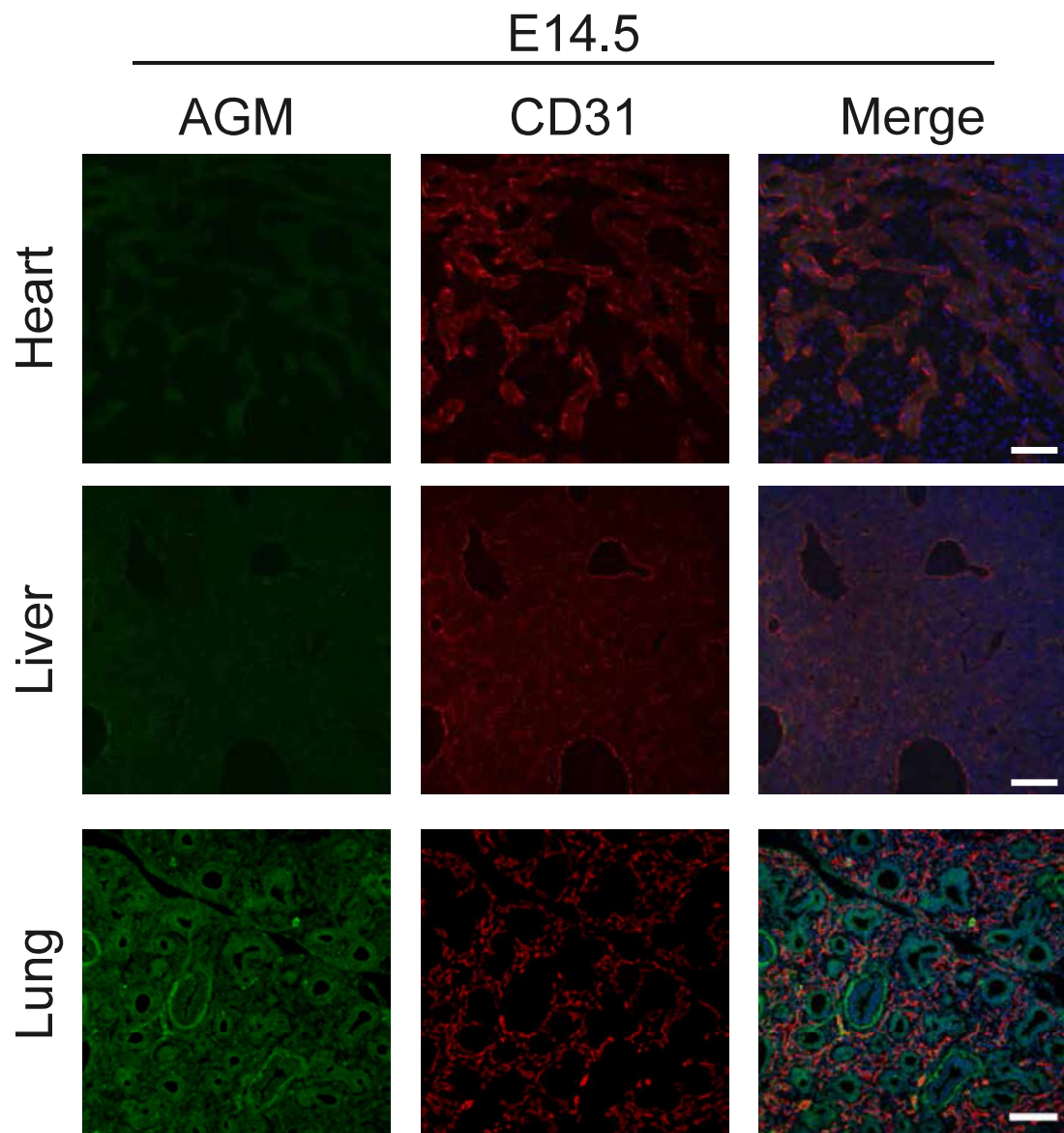

Supplement: Supplementary file 1 — Additional file 1: Figure S1. Absence of AGM expression in capillaries of peripheral organs of adult mice. In the heart (upper panel) we could detect some AGM staining (green) only in large vessels, and in the liver (lower panel), low AGM staining could be detected in a small fraction of non-vascular cells (presumably hepatocytes, arrowheads) but not in the vasculature. N = 3 mice; scale bar 50 µm. Figure S2. Absence of AGM expression in capillaries of developing peripheral organs. As in E12.5, AGM expression was specific to CNS vasculature also in E14.5, as it was not detected in developing vasculature of peripheral organs such as the heart, liver or lung. N = 3 mice; scale bar 100 µm (upper and middle panel) and 50 µm (lower panel). [file 12987_2020_188_MOESM1_ESM.pdf]
